# Low plasma magnesium concentration and future abdominal aortic calcifications in moderate chronic kidney disease

**DOI:** 10.1186/s12882-021-02267-4

**Published:** 2021-02-25

**Authors:** Anique D. ter Braake, Larissa P. Govers, Mieke J. Peeters, Arjan D. van Zuilen, Jack F. M. Wetzels, Peter J. Blankenstijn, Joost G. J. Hoenderop, Jeroen H. F. de Baaij, Jan A. J. G. van den Brand, For the MASTERPLAN study group

**Affiliations:** 1 Department of Physiology, Radboud Institute for Molecular Life Sciences, Radboud University Medical Center, Nijmegen, The Netherlands; 2 Department of Nephrology, Radboud Institute for Health Sciences, Radboud University Medical Center, PO box 9101, 6500 HB Nijmegen, The Netherlands; 3 Department of Nephrology, University Medical Center Utrecht, Utrecht, The Netherlands

**Keywords:** Abdominal aortic calcification score, Chronic kidney disease, Magnesium, Vascular calcification

## Abstract

**Background:**

Higher plasma magnesium concentrations are associated with reduced cardiovascular disease risk in chronic kidney disease (CKD) patients. The importance of plasma magnesium concentration for vascular calcification in earlier stages of CKD remains underexplored. This study investigated whether plasma magnesium is a determinant for the presence and severity of vascular calcification in moderate CKD.

**Methods:**

Retrospective analysis was performed using abdominal aortic calcification (AAC) scores in 280 patients with stage 3 and 4 CKD enrolled in the MASTERPLAN trial. Lateral abdominal X-ray was used to evaluate AAC. Plasma magnesium concentration were measured over time. A zero-inflated Poisson model determined the association between plasma magnesium concentration and AAC.

**Results:**

79 out of 280 patients did not have AAC, and in patients with AAC the median calcification score was 3.5 (interquartile range: 0.0–8.6). The mean plasma magnesium concentration was 0.76 ± 0.10 mmol/L at baseline. A 0.1 mmol/L higher plasma magnesium concentration was associated with lower AAC of 0.07 point (95% CI -0.28 – 0.14). A 0.1 mmol/L higher plasma magnesium lowered the odds of detecting any AAC by 30% (OR = 0.63; 95% CI 0.29–1.37). After 1 year and 4 years (at time of X-ray) of follow-up this association was attenuated (OR = 0.93; 95% CI 0.61–1.43 and 0.93; 95% CI 0.60–1.45, respectively). None of these associations reached statistical significance.

**Conclusions:**

Plasma magnesium concentration at baseline is not associated with the risk for future AAC. Interventions increasing magnesium to avoid vascular calcification may have greatest potential in early CKD stages prior to onset of vascular calcification.

**Supplementary Information:**

The online version contains supplementary material available at 10.1186/s12882-021-02267-4.

## Background

In patients with chronic kidney disease (CKD) cardiovascular complications are the main cause of mortality [1, 2]. These cardiovascular complications are often a consequence of vascular calcification, which occurs in 80% of CKD patients with end-stage disease [3]. Vascular calcification is provoked by disturbances in mineral-bone metabolism in CKD, mainly characterized by hyperphosphatemia [4]. Currently, there is no effective treatment for vascular calcification. Presently used methods aimed at lowering blood phosphate (Pi) concentrations are insufficient to limit vascular calcification or cardiovascular disease risk [5]. Over the past decade, magnesium (Mg^2+^) has gained attention as a potential modifiable risk factor of vascular calcification in CKD [6]. Indeed, recent data demonstrate that magnesium prevents the formation of secondary calciprotein particles, which contribute to the development of medial calcification. Phosphate is the major determinant of secondary calciprotein particle formation and explains why CKD patients are prone to the development of these particles. Magnesium is a protective factor in the calcification milieu, which may act as a phosphate-buffering system to prevent secondary calciprotein particle development [7].

Increased plasma Mg^2+^ is associated with reduced risk for all-cause and cardiovascular mortality in the general population and in CKD patients [8–15]. More specifically, Mg^2+^ effectively prevents vascular calcification in human vascular smooth muscle cells as well as in a variety of rodents [16–18]. A recent clinical trial in CKD patients reported immediate effects of increasing both oral and dialysate Mg^2+^ on calcification propensity of human serum, as measured by in-vitro analysis [19–21]. Combined, these studies demonstrate that increasing plasma Mg^2+^ concentrations reduces vascular calcification risk and progression in end-stage CKD patients. Until now, most observational cohort studies on which clinical trials are based have focused on the association between Mg^2+^ and vascular calcification in hemodialysis patients. However, the potential importance of plasma Mg^2+^ concentration for vascular calcification in earlier stages of CKD remains underexplored.

The aim of this study was to investigate whether plasma Mg^2+^ is a determinant for the presence and severity of vascular calcification in moderate CKD. We performed a retrospective study using abdominal aortic calcification (AAC) scores in patients with stage 3 and 4 CKD that were enrolled in the MASTERPLAN (Multifactorial Approach and Superior Treatment Efficacy in Renal Patients with the Aid of Nurse practitioners) trial [22].

## Methods

### Design and patient inclusion

A comprehensive description of patient selection and the assessment of AAC has been previously reported by Peeters et al. [23] For the reader’s convenience we will briefly summarize the approach. The MASTERPLAN (Multifactorial Approach and Superior Treatment Efficacy in Renal Patients with the Aid of Nurse practitioners) study was a randomized controlled trial that started inclusion in 2004 (﻿ISRCTN73187232). Rationale, design and outcomes are reported elsewhere [22, 24, 25]. In summary, the MASTERPLAN trial was designed as a multifactorial intervention comparing additional renal nurse support to standard care to reduce cardiovascular and renal risk in patients with moderate CKD. Adult patients with moderate to severe CKD (estimated creatinine clearance between 20 and 70 ml/min/1.73m^2^) were included [22]. Patients with a renal transplant < 1 year before screening, acute kidney injury or rapidly progressing glomerular nephritis, any malignancy < 5 years before screening (other than basocellular or squamous cell carcinoma of the skin) or participating in other clinical trials that required the use of study medication were excluded from the study [22]. The study was performed in accordance with the declaration of Helsinki. All patients provided written informed consent, and medical ethical approval was obtained prior to initiation of the MASTERPLAN trial. In the period of 2008–2009 nephrologists considered to role of evaluating AAC in selected patients, based on the data and discussions that resulted in the recommendation in the 2009 KDIGO CKD-MBD guideline [23, 26]. As the X-ray was not in the initial trial protocol, the decision to take X-rays was left to the treating nephrologist. In total 280 patients had an X-ray. The lateral abdominal X-rays were reviewed by two independent reviewers.

### Assessment of abdominal aortic calcification

The presence of AAC was determined and scored according to the method described by Kauppila et al. [27] This calcification score takes into account the anterior and the posterior arterial all separately, and ranges from 0 to 24. A detailed description of the calcification grading in the MASTERPLAN study has been documented previously [23]. The interrater agreement was excellent with a linear weighted kappa of 0.87. In only 8 of the 2240 rated aorta segments the score deviated > 1 point.

### Plasma magnesium measurements

Plasma Mg^2+^ concentrations were determined for all patients in mmol/L using a colorimetric assay according to the manufacturer’s protocol (Roche, Basel, Switzerland) and measured at 600 nm on a Bio-Rad Benchmark plus microplate reader (Bio-Rad Laboratories, Hercules, California, USA). Plasma samples had been frozen and stored at − 80° prior to analysis. All measurements were performed in triplicate. Plasma Mg^2+^ concentrations were measured at baseline, after one year and at the time of the X-ray (after four years).

### Statistical analyses

Baseline data were described by frequencies and proportion for categorical variables, mean and standard deviation (SD) for normally distributed continuous variables, and median and interquartile range for continuous variables with a skewed distribution. We investigated the dose response relation between AAC score and serum Mg^2+^ by creating a scatterplot and fitting a LOESS smoothed regression line. As the association between AAC and plasma Mg^2+^ was approximately linear, no transformations were considered. Next, we reviewed missing data patterns (Supplementary Table S1) and used multiple imputation with chained equations to impute missing values using R-package ‘mice’ [28]. Predictive mean matching was used to impute missing values for continuously distributed variables and logistic regression was used to impute missing values for dichotomous variables. For all imputation models, predictors with a bivariate correlation of > 0.15 were considered. Diagnostic plots indicated that the imputations were stable over five iterations. Strip plots showed that imputed values all fell within the range of the observed values and were distributed across the entire range of observed values (Supplementary Figure S1).

In order to obtain a valid estimate for the association between plasma Mg^2+^ and AAC a multivariate model that adjusts for important confounders is required. To identify the variables that should be adjusted for, a causal model with the hypothesized relation between plasma Mg^2+^ and AAC was created. A directed acyclic graph was created with dagitty.net software and associated R-package to encode model assumptions [29]. Implied conditional independencies stemming from the model were tested and the model was refined until no gross violations were detected (see Supplementary Table S2 for the results of the conditional independency tests and Supplementary Fig. S2 for the final causal model). We arrived at two possible adjustment sets (Table 2). Both were used to obtain an adjusted estimate for the association between plasma Mg^2+^ concentration and AAC.

As 79 of the 280 patients had no calcifications we used a zero-inflated Poisson model to determine the association between plasma Mg^2+^ concentration and AAC. The model assumes that the zeros are generated by another process than the count data, and therefore that these processes can be modeled separately. The model consists of two parts. First, a Poisson model for the continuous data with values > 0, and second a logistic model that estimates the log-odds of a zero observation. Additionally, we included an off-set for the time between Mg^2+^ measurement and the X-ray in the Poisson sub-model.

All analyses were performed with RStudio (version 1.1.463 –© 2009–2018 RStudio, Inc.), R (version 3.5.3 for Windows) and the following packages: dagitty_0.2–3, boot_1.3–20, mice_3.5.0, V8_2.2, car_3.0–2, survival_2.44–1.1, tableone_0.10.0, ggplot2_3.1.0, dplyr_0.8.1, foreign_0.8–71 (Item S1) [30].

## Results

### Patient population

Table 1 shows the baseline characteristics of the 280 patients included in the analysis [23]. This cohort consisted of relatively young patients with a mean age of 61 years. With an average eGFR of 41 ml/min/1.73m^2^ patients had moderately to severely decreased renal function. In total, 79 out of 280 patients did not have AAC (AAC = 0) and the median calcification score was 3.5 (interquartile range: 0.0–8.6) [21]. The majority of the included patients was diagnosed with a hypertensive or renovascular cause of CKD. Only 10% of the patients had diabetic nephropathy as cause of the CKD, while 23% of the patients had diabetes. Around 30% of the patients had cardiovascular disease at time of inclusion. In addition, Pi and fibroblast growth factor 23 (FGF-23) concentrations fell within the normal range and did not differ between patients with or without AAC.

**Table 1:** Baseline characteristics

	Total	No AAC	AAC	***P***
*n*	*280*	*79*	*201*	
Randomized to intervention group	164 (58.6)	46 (58.2)	118 (58.7)	1.00
Female gender	88 (31.4)	28 (35.6)	60 (29.9)	0.45
Age (years)	61.0 [51.7, 68.0]	49.0 [39.5, 60.0]	64.0 [57.0, 70.0]	< 0.001
*Race*				
Caucasian	251 (89.6)	68 (86.1)	183 (91.0)	
Non-Caucasian	29 (10.4)	11 (13.9)	18 (9.0)	
*Diagnosis*^a^				0.04
Diabetic Nephropathy	30 (10.7)	5 (6.3)	25 (12.4)	
Renovascular	87 (31.1)	17 (21.5)	70 (34.8)	
Glomerulonephritis	53 (18.9)	20 (25.3)	33 (16.4)	
Interstitial Nephritis	30 (10.7)	13 (16.5)	17 (8.5)	
Congenital	25 (8.9)	9 (11.4)	16 (8.0)	
Unknown	55 (19.6)	15 (19.0)	40 (19.9)	
Diabetes^b^	64 (22.9)	9 (11.4)	55 (27.4)	0.01
CVD^c^	81 (28.9)	10 (12.7)	71 (35.3)	< 0.001
Current smoker	52 (18.6)	13 (16.5)	39 (19.5)	0.68
BMI (kg/m^2^)	26.0 [23.6, 28.0]	24.6 [23.1, 26.5]	26.5 [24.2, 28.4]	< 0.001
Waist hip ratio	0.95 (0.08)	0.94 (0.08)	0.96 (0.08)	0.04
SBP (mmHg)	133 (20)	128 (17)	135 (21)	0.01
DBP (mmHg)	77 (11)	78 (10)	77 (12)	0.21
eGFR^d^ (mL/min per 1.73m^2^)	41.8 (19.0)	43.1 (20.2)	41.3 (18.5)	0.48
Serum creatinine (μmol/L)	161.3 [129.9, 198.8]	163.8 [133.4, 195.3]	160.8 [127.9, 201.8]	0.72
Serum albumin (g/dL)	40.5 (3.7)	41.0 (4.0)	40.3 (3.5)	0.15
Total serum cholesterol (mmol/L)	4.89 (1.08)	4.99 (1.15)	4.85 (1.05)	0.33
LDL cholesterol (mmol/L)	2.83 (0.98)	2.87 (1.09)	2.81 (0.94)	0.64
Hemoglobin (mmol/L)	8.3 (0.9)	8.2 (1.0)	8.4 (0.9)	0.09
Ca^2+^ (mmol/L)	2.38 (0.14)	2.39 (0.16)	2.37 (0.13)	0.23
Mg^2+^ (mmol/L)	0.76 (0.10)	0.73 (0.10)	0.77 (0.09)	0.01
Pi (mmol/L)	1.07 [0.94, 1.21]	1.07 [0.92, 1.18]	1.08 [0.94, 1.24]	0.20
FGF-23 (RU/L)	100.3 [58.8, 166.7]	91.1 [51.4, 161.2]	108.0 [64.0, 168.0]	0.18
PTH (pmol/L)	8.1 [5.2, 12.6]	8.3 [5.4, 14.1]	8.0 [5.2, 12.1]	0.52
hsCRP (mg/dL)	2.0 [0.8, 51.3]	1.7 [0.6, 4.3]	2.1 [1.0, 5.6]	0.09
Proteinuria (g/24 h)	0.2 [0.1, 0.6]	0.2 [0.1, 0.6]	0.2 [0.1, 0.6]	0.28
ACEi/ARB use	239 (85.4)	62 (78.5)	177 (88.1)	0.06
Diuretic use	137 (48.9)	31 (39.2)	106 (52.7)	0.06
Other antihypertensives	157 (56.1)	34 (43.0)	123 (61.2)	0.01
Lipid lowering drugs	204 (72.9)	46 (58.2)	158 (78.6)	0.001
Vitamin D use	49 (17.5)	16 (20.3)	33 (16.4)	0.56
Phosphate binder use	25 (8.9)	8 (10.1)	17 (8.5)	0.84
AAC score	3.50 [0.00, 8.62]	0.00 [0.00, 0.00]	6.50 [3.00, 10.50]	

Studied by logistic regression. Data are given as number (%), mean (SD) or median [interquartile range]

*AAC* Abdominal aortic calcification, *ACEi* Angiotensin converting enzyme inhibitor, *ARB* Angiotensin-II receptor blockers, *BMI* Body mass index, *Ca*^*2+*^ Calcium (conversion factor /0.2495 for mg/dL), *CVD* Cardiovascular disease, *DPB* Diastolic blood pressure, *eGFR* Estimated glomerular filtration rate, *FGF-23* Fibroblast growth factor 23, *hsCRP* High-sensitivity C-reactive protein, *LDL* Low-density lipoprotein (conversion factor cholesterol /0.02586 for mg/dL), *Mg*^*2+*^ Magnesium, *Pi* Phosphate (conversion factor /0.3229 for mg/dL), *PTH* Parathyroid hormone, *SBP* Systolic blood pressure. Creatinine conversion factor /88.4 for mg/dL

^a^Diagnosis of the underlying renal disease was determined by the treating physician using available patient history, clinical course and if available histopathology

^b^Diabetes was defined as using blood glucose lowering medication or fasting glucose > 7.0 mmol/L

^c^Cardiovascular disease was defined as myocardial infarction, stroke or vascular intervention

^d^Using the MDRD (modification of diet in renal disease) eq. (186 x (Creatinine/88.4)^-1.154^ x (Age)^-0.203^ x (0.742 if female) x (1.210 if black), re-expressed for standardized serum creatinine

### Distribution of plasma magnesium concentrations

**Fig. 1:**
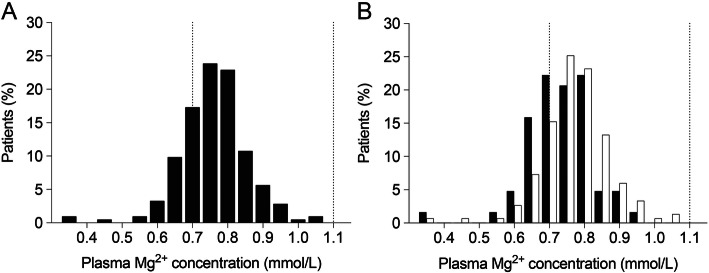
Plasma Mg^2+^ concentrations in non-dialysis CKD patients. Distribution of Mg^2+^ concentrations (A). Plasma Mg^2+^ concentrations for CKD patients with (indicated in white) and without (indicated in black) abdominal aortic calcifications (B). Dotted vertical lines indicate the reference values for Mg^2+^ concentration (0.7–1.1 mmol/L)

For all 280 patients, plasma Mg^2+^ concentration was measured at baseline, after one year and after four years. After a median period of 3.7 (interquartile range: 3.1–4.0) years after baseline, the X-rays were performed [21]. The mean plasma Mg^2+^ concentration was 0.76 ± 0.10 mmol/L at baseline (Fig. 1A). Patients without AAC appeared to have somewhat higher plasma Mg^2+^ concentrations at baseline (Fig. 1B). No marked differences in plasma Mg^2+^ concentrations were observed between baseline, after one year (0.76 ± 0.10 mmol/L) and at time of X-ray (0.74 ± 0.10 mmol/L). The lowest and the highest Mg^2+^ concentration were measured at 0.35 and 1.05 mmol/L, respectively. Approximately 16% of the patients had hypomagnesaemia with a plasma Mg^2+^ concentration below 0.7 mmol/L.

### Dose-response relation between magnesium and AAC

**Fig. 2:**
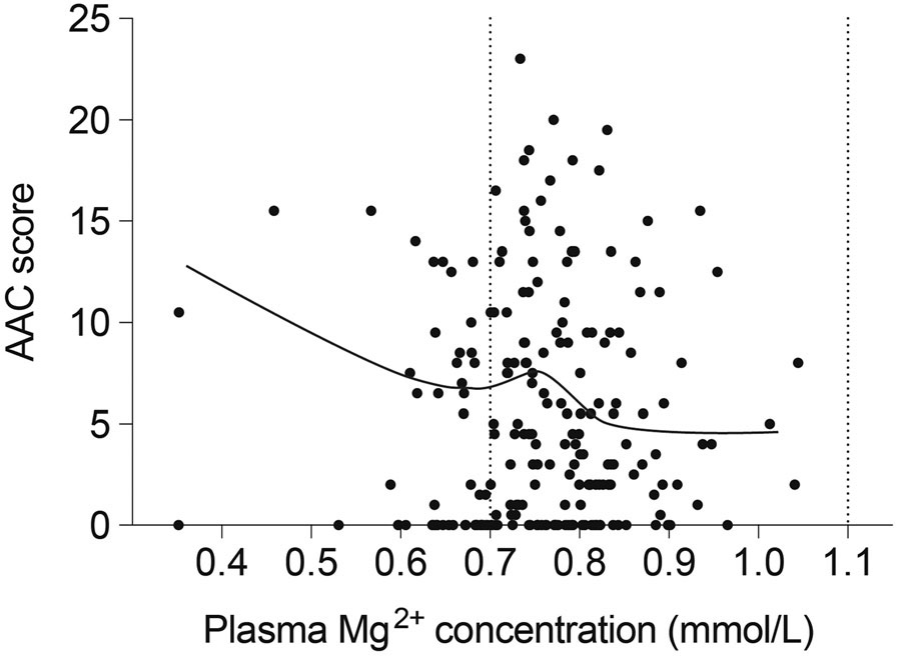
Dose-response relation between Mg^2+^ and abdominal aortic calcification score. The abdominal aortic calcification (AAC)-score for each patient are presented individually and plotted against their plasma Mg^2+^ concentration at baseline. Dose-response relation between AAC and plasma Mg^2+^ concentration was investigated by creating a scatter plot and fitting a LOESS smoothed regression line. No transformations were considered due to linearity of the association. Dotted vertical lines indicate the reference values for Mg^2+^ concentration (0.7–1.1 mmol/L)

The dose-response relationship between plasma Mg^2+^ concentration and the AAC score determined from X-rays taken three to four years later is shown in Fig. 2. The crude Poisson model demonstrated that a 0.1 mmol/L higher plasma Mg^2+^ concentration was associated with a 0.07 point lower value of AAC (95% Confidence Interval (CI) -0.28 – 0.014). The crude logistic model showed that a 0.1 mmol/L higher baseline plasma Mg^2+^ concentration resulted in 30% lower odds for detecting any AAC three to four years later (OR = 0.63; 95% CI 0.29 to 1.37). The association between AAC at year three or four and Mg measurement at year one n was attenuated compared to baseline to 7% per 0.1 mmol/L increase in plasma Mg^2+^ (OR = 0.93; 95% CI 0.61–1.43). Likewise, when Mg^2+^ measurements were taken at the same time as the X-ray the odds of absence of a calcification were 7% per 0.1 mmol/L Mg^2+^ increase (OR = 0.93; 95% CI 0.60–1.45). Adjustment did not substantially change this association (Table 2). None on the association described above reached statistical significance, as can be determined from the 95% confidence intervals overlapping 1.0.

**Table 2:** Associations between AAC and plasma Mg^2+^ in multivariate analysis

AAC incidence	*Per 0.1 mmol/L Mg*^*2+*^	*95% CI*
*Univariate analysis*		
Baseline		
Count (Poisson model)	−0.07	−0.28 – 0.14
Odds Ratio (zero model)	0.63	0.29–1.37
1 year		
Count (Poisson model)	−0.08	− 0.51 – 0.35
Odds Ratio (zero model)	0.93	0.61–1.43
X ray		
Count (Poisson model)	−0.08	−0.51 – 0.35
Odds Ratio (zero model)	0.93	0.60–1.45
*Adjusted for age, calcium, phosphate, cardiovascular disease, and diabetes*		
Baseline		
Count (Poisson model)	−0.07	−0.58 – 0.44
Odds Ratio (zero model)	0.63	0.29–1.37
1 year		
Count (Poisson model)	−0.08	− 0.51 – 0.35
Odds Ratio (zero model)	0.93	0.61–1.43
X ray		
Count (Poisson model)	−0.08	− 0.51 – 0.35
Odds Ratio (zero model)	0.93	0.60–1.45
*Adjusted for age, calcium, phosphate, cardiovascular disease, eGFR, and PTH*		
Baseline		
Count (Poisson model)	−0.07	−0.53 – 0.39
Odds Ratio (zero model)	0.64	0.32–1.30
1 year		
Count (Poisson model)	−0.07	− 0.45 – 0.32
Odds Ratio (zero model)	0.94	0.63–1.38
X ray		
Count (Poisson model)	-0.07	− 0.44 – 0.31
Odds Ratio (zero model)	0.93	0.63–1.38
*Adjusted for age, eGFR, phosphate, and diuretics*		
Baseline		
Count (Poisson model)	−0.08	−0.53 – 0.38
Odds Ratio (zero model)	0.65	0.34–1.23
1 year		
Count (Poisson model)	-0.07	− 0.48 – 0.35
Odds Ratio (zero model)	0.93	0.61–1.41
X ray		
Count (Poisson model)	−0.07	−0.48 – 0.33
Odds Ratio (zero model)	0.94	0.63–1.40

A zero-inflated Poisson model was used to determine the association between plasma Mg^2+^ concentration and AAC. The model assumes that the zeros are generated by another process than the count data, and therefore that these processes can be modeled separately. The model consists of two parts. First, a Poisson model for the continuous data with values > 0 and second, a logistic model that estimates the Log-odds of a zero observation

Results are presented per 0.1 mmol/L increase in plasma Mg^2+^

*Ca*^*2+*^ Calcium, *CI* Confidence interval, *Mg*^*2+*^ Magnesium

## Discussion

In this study, we aimed to investigate whether plasma Mg^2+^ concentration is a determinant for the presence and severity of vascular calcification in moderate CKD. We have identified a modest, not statistically significant association between AAC score and plasma Mg^2+^ concentration. For every higher value of 0.1 mmol/L plasma Mg^2+^ the associated AAC score is lower by approximately 0.1 point. In addition, a more pronounced association was present between higher plasma Mg^2+^ concentration at baseline and the absence of AAC four years later. Specifically, the odds of finding any AAC on the X-ray after four years are 30% lower per 0.1 mmol/L higher value in plasma Mg^2+^ concentration at baseline. The observed association weakens markedly after one year (shorter period before the X-ray) and is almost absent when plasma Mg^2+^ concentration is measured at time of the X-ray.

Other studies have reported stronger associations between plasma or serum Mg^2+^ concentration and calcification score [9, 10, 31–35]. Molnar et al. showed that a 0.1 mmol/L higher serum Mg^2+^ concentration was associated with a 1.1-point lower AAC score in end-stage renal disease patients [34]. In pre-dialysis CKD patients, every mg/dL (0.4 mmol/L) higher serum Mg^2+^ concentration was associated with a 0.36 point lower CAC-density score (scale 1–3.5) [33]. Interestingly, Sakaguchi et al. described that the association between Mg^2+^ and AAC in pre-dialysis patients is dependent on serum Pi concentration. This association was only identified in a sub-group where serum Pi concentration was above 1.1 mmol/L, but not in patients with a Pi concentration below 1.1 mmol/L. [33] In the MASTERPLAN cohort, median plasma Pi concentration was 1.07 mmol/L. In other studies describing an association between Mg^2+^ concentration and AAC, the mean serum Pi concentration exceeded 1.49 mmol/L (Table 3). The relatively low Pi concentration potentially related to CKD stage may explain the weak correlation between Mg^2+^ concentration and AAC score found in our study. Of note, with a plasma Mg^2+^ concentration of 0.76 ± 0.10 mmol/L, plasma Mg^2+^ concentrations were low in comparison to other studies (Table 3). These relatively low Mg^2+^ concentrations, in addition to the low variation, could be a reason for the absence of a stronger relationship between plasma Mg^2+^ concentration and AAC score. Moreover, the average Mg^2+^ concentrations at baseline was higher in the AAC group. However, in the inferential analysis we accounted for other possible determinants of AAC. These include diabetes, higher age, prior cardiovascular and serum calcium and phosphate. Diabetes prevalence, age, and CVD prevalence were substantially higher in the AAC group, while serum calcium and phosphate concentrations were similar. The crude association between Mg^2+^ and AAC in our study, may thus be explained by these confounding factors However, absence of evidence does not mean evidence of absence. We hypothesize that the absence of a relation in our cohort may have two reasons: First the serum Mg^2+^ concentrations were fairly low, possibly insufficient to achieve and effect on AAC. Second, Mg^2+^ may not have a (clinically meaningful) effect on established AAC. By design we could ascertain if patients had AAC at baseline already. Of note, the plasma Mg^2+^ concentrations for each patient were stable between the measurements. In advanced CKD, serum Mg^2+^ concentrations tend to increase, reaching values of around 0.97 mmol/L. [13] In addition, the median AAC score in this study was 3.5, while in stage 5 CKD mean AAC was 8.9 (scale 0–24) [34]. Our study population included patients with moderate CKD, with most in stage 3b. It is possible that a more pronounced association between Mg^2+^ concentration and AAC is present in more advanced stages of CKD.

**Table 3:** Overview of studies assessing the relationship between blood Mg^2+^ concentration and vascular calcification

Reference^**#**^	Study type	CKD stage	Sample size (% women)	Mg^**2+**^ concentration	Pi concentration	Type	Follow-up (years)	Association (***P*** < 0.05)	Associations serum Mg^**2+**^ concentration (mmol/L)
Meema et al. 1987 [9]	Prospective	5	44 (0)	1.16	1.88	Peripheral AC	2	Yes	1.10 ± 0.21 in AC compared to 1.24 ± 0.21 in non-AC
Tzanakis et al. 1997 [36]	Cross-sectional	5	56 (39)	1.23	1.63	MAC	–	Yes	1.14 ± 0.12 in MAC versus 1.27 ± 0.10 in non-MAC
Ishimura et al. 2007 [37]	Cross-sectional	5	390 (42)	1.14	1.87	AC (hand)	–	Yes	1.10 ± 0.12 in VC versus 1.14 ± 0.14 in non-VC
Matias et al. 2014 [32]	Prospective	5	206 (45)	1.36	1.49	SVCS	4	Yes	β-coefficient 0.17 95% CI 0.08–0.30 (cut-off concentration 1.15)^1^
Sakaguchi et al. 2016 [33]	Cross-sectional	3–4	109 (33)	0.85	≥ 1.10	CAC	–	Yes	β-coefficient − 0.36 (CI not reported)^2^
Molnar et al. 2017 [34]	Cross-sectional	5	80 (30)	0.84	1.70	AAC	–	Yes	Adjusted R^2^ 0.18, β-coefficient − 12.27, 95% CI − 19.54 – − 5.00^3^
Okamoto et al. 2018 [35]	Retrospective	5	128 (36)	0.90	1.74	AAC	1	Yes	OR 3.11, 95% CI 1.43–5.89 (baseline serum Mg^2+^ < 0.9)
Tamura et al. 2019 [38]	Prospective	5	392 (34.7)	1.15	1.65	AoAC	4.2	No	–

*(A/C)AC, (abdominal/coronary) arterial calcification; AoAc, aortic arch calcification; MAC, mitral annular calcifications; OR, odds ratio; SVCS, simple vascular calcification score (hands/pelvis); VC, vascular calcification

^#^Articles were obtained after PubMed search in October 2019 using the following search terms: ((“Renal insufficiency, Chronic”[Mesh] OR “Chronic kidney disease”[TiAb]) AND “Magnesium”[Mesh/TiAb]) AND (“calcinosis”[mesh] OR “calcification”[TiAb])

^1^Scale of calcification score reported was not quantitative [39]. Score is based on presence in pre-determined locations and scores are made up out of the sum of positive locations, ranging from 0 to 8 [36]

^2^Scale of calcification density was reported between 0.86–3.33 (Agatston score divided by the total calcified area for each patient)

^3^Scale of calcification score (AAC) was reported 0–24

Our results suggest that a 0.1 mmol/L higher value of plasma Mg^2+^ concentration is associated with 30% reduced risk of having any AAC, although not statistically significantly. Because vascular calcification is irreversible once established, determining the optimal window of effective treatment, potentially using Mg^2+^, is essential [40]. To date, most epidemiological studies have investigated whether plasma Mg^2+^ concentration is associated with vascular calcification in dialysis patients where calcification has already progressed (Table 3) [9, 31, 32, 34, 37]. Vascular calcification often manifests already in earlier stages of CKD. Our results indicate that, at least in this cohort consisting of 280 non-dialysis CKD patients, the effects of Mg^2+^ may be lagged. Therefore, supplementation of Mg^2+^ may be less effective once AAC has already formed. This notion is in line with the study of Bressendorff et al, showing that an increase in blood Mg^2+^ concentration of 0.34 mmol/L results in reduced calcification propensity, which reflects a lower Ca^2+^-Pi precipitation risk [19]. The calcification propensity test determines the formation of calcium phosphate particles in human serum in an in vitro setup. Thus, the study of Bressendorff determines the ex vivo formation of calcium precipitates, rather than measuring already formed calcification. As such, the study supports our data and demonstrates that magnesium may prevent the formation of calcification, but will not affect calcification already in place.

A vast body of observational studies has identified associations between the blood Mg^2+^ concentration and cardiovascular and all-cause mortality in end-stage renal disease patients [6, 11, 13]. While subsequent in vitro and animal study evidence has been compelling, clinical studies assessing the effectiveness of Mg^2+^ in preventing vascular calcification have been scarce [16–18, 41–43]. Recently, in a randomized clinical study oral Mg^2+^ supplementation resulted in diminished progression of coronary artery calcification (CAC)-score in pre-dialysis CKD patients [44]. While more clinical studies are currently underway, it is of importance to evaluate the most effective window of intervention, which is likely in early CKD before onset of vascular calcification.

Strengths of our study include the use of a well characterized study cohort that has been followed-up according to standardized procedures and with extensive biobanking. In addition, we used the state-of-the-art methodology for causal inference to create an explicit and testable causal model. Furthermore, plasma Mg^2+^ concentrations were obtained at several time points which allowed for the determination of the latency of the protective effect of plasma Mg^2+^ concentration on vascular calcification. The calcifications were scored with high interrater reliability, reducing the possibility of misclassification.

A major limitation of our study was the relatively small sample size and the fact that a lumbar X-ray was only available in a subgroup of patients. A selection bias may have been created by selecting patients that received the X-ray that were relatively healthy. Although the differences were small, patients that received the X-ray had a lower renal risk and a higher cardiovascular risk profile. Moreover, patients in the MASTERPLAN trial were fairly well controlled both at baseline and follow-up. Therefore, this population may not be completely representative of the average CKD population. Furthermore, some variables had missing values, mostly during follow-up. Missingness was handled by using multiple imputation. However, while this approach reduces the likelihood of selection bias, it does introduce noise in the covariate values. As a consequence, residual confounding may remain despite statistical adjustment. Furthermore, as already discussed for the MASTERPLAN cohort study in a previous publication, the sensitivity of AAC measurements by lumbar X-ray is less compared to computed tomography measurements, leading to a potential underestimation of AAC severity [22, 23]. This could lead to misclassification of patients without AAC. Another limitation is the lack of X-ray data at baseline of the study, thus possible effects of Mg^2+^ on the rate of calcification in patients with established calcifications could not be investigated. Finally, we did not have information about the types of phosphate binding medication that patients used. Some of these may include magnesium salts..

## Conclusions

In conclusion, a statistically nonsignificant association between Mg^2+^ and AAC in this study suggests a limited if any potential preventive effect Mg^2+^ on the development of AAC in non-dialysis CKD patients.

## Supplementary Information

**Additional file 1: Table S1.** Missingness patterns at baseline. **Table S2.** Conditional independency tests for the causal assumption model. **Figure S1.** Diagnostic strip plots. **Figure S2.** Causal model for identification of adjustment sets. Item S1 Supplementary R-code.

## Data Availability

Original study data and associated analysis scripts have been stored in a virtual environment on the anDREa platform (https://www.andrea-consortium.org/). Access can be requested via the corresponding author.

## Abbreviations

AAC: Abdominal aortic calcification
CAC: Coronary Artery Calcification
CKD: Chronic Kidney Disease
KDIGO CKD-MBD: Kidney Disease Improving Global Outcomes Chronic Kidney Disease – Mineral Bone Disease
MASTERPLAN: Multifactorial Approach and Superior Treatment Efficacy in Renal Patients with the Aid of Nurse practitioners trial
Mg^2+^: Magnesium
LOESS: Locally estimated sum of squares
OR: Odds ratio
SD: Standard Deviation
