# Supplementary material for: Low plasma magnesium concentration and future abdominal aortic calcifications in moderate chronic kidney disease
Source: BMC Nephrol. 2021 Feb 25;22:71. doi: 10.1186/s12882-021-02267-4 (PMC7905862; doi:10.1186/s12882-021-02267-4)
Supplement: Supplementary file 1 — Additional file 1: Table S1. Missingness patterns at baseline. Table S2. Conditional independency tests for the causal assumption model. Figure S1. Diagnostic strip plots. Figure S2. Causal model for identification of adjustment sets. Item S1 Supplementary R-code. [file 12882_2021_2267_MOESM1_ESM.docx]

**SUPPLEMENTARY MATERIAL**

**SUPPLEMENTARY TABLES**

**Table S1 Missingness patterns at baseline**

| **Count** | **Missing data** | | | | | | | | | | | | | |
| --- | --- | --- | --- | --- | --- | --- | --- | --- | --- | --- | --- | --- | --- | --- |
|  | Smoke | Waist | Hip | WHR | HDL | PTH | LDL | CHD | Stroke | uProt | FGF-23 | CysC | hsCRP | Mg^2+^ |
| 146 | 1 | 1 | 1 | 1 | 1 | 1 | 1 | 1 | 1 | 1 | 1 | 1 | 1 | 1 |
| 34 | 1 | 1 | 1 | 1 | 1 | 1 | 1 | 1 | 1 | 1 | 1 | 1 | 1 | 0 |
| 5 | 1 | 1 | 1 | 1 | 1 | 1 | 1 | 1 | 1 | 1 | 1 | 1 | 0 | 1 |
| 1 | 1 | 1 | 1 | 1 | 1 | 1 | 1 | 1 | 1 | 1 | 1 | 0 | 1 | 0 |
| 19 | 1 | 1 | 1 | 1 | 1 | 1 | 1 | 1 | 1 | 1 | 1 | 0 | 0 | 1 |
| 5 | 1 | 1 | 1 | 1 | 1 | 1 | 1 | 1 | 1 | 1 | 1 | 0 | 0 | 0 |
| 15 | 1 | 1 | 1 | 1 | 1 | 1 | 1 | 1 | 1 | 1 | 0 | 1 | 1 | 1 |
| 3 | 1 | 1 | 1 | 1 | 1 | 1 | 1 | 1 | 1 | 1 | 0 | 1 | 1 | 0 |
| 2 | 1 | 1 | 1 | 1 | 1 | 1 | 1 | 1 | 1 | 1 | 0 | 0 | 0 | 1 |
| 16 | 1 | 1 | 1 | 1 | 1 | 1 | 1 | 1 | 1 | 1 | 0 | 0 | 0 | 0 |
| 11 | 1 | 1 | 1 | 1 | 1 | 1 | 1 | 1 | 1 | 0 | 1 | 1 | 1 | 1 |
| 1 | 1 | 1 | 1 | 1 | 1 | 1 | 1 | 1 | 1 | 0 | 1 | 1 | 1 | 0 |
| 1 | 1 | 1 | 1 | 1 | 1 | 1 | 1 | 1 | 1 | 0 | 0 | 0 | 0 | 0 |
| 7 | 1 | 1 | 1 | 1 | 1 | 1 | 1 | 0 | 0 | 1 | 1 | 1 | 1 | 1 |
| 2 | 1 | 1 | 1 | 1 | 1 | 1 | 1 | 0 | 0 | 1 | 1 | 1 | 1 | 0 |
| 2 | 1 | 1 | 1 | 1 | 1 | 1 | 1 | 0 | 0 | 1 | 1 | 0 | 0 | 1 |
| 2 | 1 | 1 | 1 | 1 | 1 | 1 | 1 | 0 | 0 | 1 | 0 | 1 | 1 | 1 |
| 1 | 1 | 1 | 1 | 1 | 1 | 1 | 1 | 0 | 0 | 1 | 0 | 1 | 0 | 0 |
| 1 | 1 | 1 | 1 | 1 | 1 | 1 | 1 | 0 | 0 | 0 | 1 | 1 | 1 | 1 |
| 1 | 1 | 1 | 1 | 1 | 1 | 1 | 0 | 1 | 1 | 1 | 0 | 0 | 0 | 0 |
| 1 | 1 | 1 | 1 | 1 | 1 | 1 | 0 | 1 | 1 | 0 | 1 | 1 | 1 | 1 |
| 1 | 1 | 1 | 1 | 1 | 1 | 0 | 1 | 1 | 1 | 1 | 1 | 1 | 1 | 1 |
| 1 | 1 | 1 | 1 | 1 | 0 | 1 | 1 | 1 | 1 | 1 | 1 | 1 | 1 | 1 |
| 1 | 1 | 0 | 0 | 0 | 1 | 1 | 1 | 1 | 1 | 0 | 1 | 1 | 1 | 1 |
| 1 | 0 | 1 | 1 | 1 | 1 | 1 | 1 | 1 | 1 | 1 | 0 | 0 | 0 | 0 |
| **Total** | 1 | 1 | 1 | 1 | 1 | 1 | 2 | 15 | 15 | 16 | 42 | 48 | 53 | 66 |

Missing data patterns were reviewed using R-package ‘mice’.^10^ Zeros indicate missing data.

CHD, coronary heart disease; CysC, cystatin C; FGF-23, fibroblast growth factor 23; HDL; high-density lipoprotein; hsCRP, high-sensitivity C-reactive protein; LDL, low-density lipoprotein; Mg^2+^, magnesium; N, number of patients; PTH, parathyroid hormone; uProt, urinary protein; WHR, waist-hip ratio.

**Table S2 Conditional independency tests for the causal assumption model**

| **Conditional Independencies** | **estimate** | **std.error** | **p.value** |
| --- | --- | --- | --- |
| aac _\|\|_ mdrd_175 \| + age + calcium + cvd + diabetes + mg + phosphate | -0.1737 | 0.2874 | 0.546 |
| aac _\|\|_ med_diur \| + age + calcium + cvd + mdrd_175 + mg + pth | 0.2332 | 0.2835 | 0.412 |
| aac _\|\|_ med_diur \| + age + calcium + cvd + diabetes + mg + phosphate | 0.1441 | 0.2833 | 0.612 |
| aac _\|\|_ pth \| + calcium + diabetes + mdrd_175 + med_diur + phosphate | -0.2530 | 0.3325 | 0.447 |
| aac _\|\|_ pth \| + age + calcium + cvd + diabetes + mg + phosphate | -0.0609 | 0.2822 | 0.829 |
| *age _\|\|_ calcium \| + mdrd_175 + med_diur* | *-2.0261* | *0.7114* | *0.005* |
| *age _\|\|_ calcium \| + cvd + mdrd_175* | *-2.1076* | *0.6905* | *0.002* |
| *age _\|\|_ med_diur \| + cvd + mdrd_175* | *1.4874* | *0.6989* | *0.034* |
| age _\|\|_ phosphate \| + diabetes + mdrd_175 | -0.4360 | 0.7306 | 0.551 |
| age _\|\|_ pth \| + mdrd_175 | -0.8152 | 0.7600 | 0.284 |
| calcium _\|\|_ cvd \| + mdrd_175 + med_diur | 0.0006 | 0.0085 | 0.939 |
| calcium _\|\|_ diabetes \| + cvd + mdrd_175 | -0.0153 | 0.0084 | 0.068 |
| calcium _\|\|_ diabetes \| + mdrd_175 + med_diur | -0.0146 | 0.0084 | 0.083 |
| calcium _\|\|_ mg \| + mdrd_175 + med_diur | 0.0023 | 0.0095 | 0.810 |
| calcium _\|\|_ phosphate \| + diabetes + mdrd_175 + pth | 0.0083 | 0.0085 | 0.334 |
| calcium _\|\|_ phosphate \| + cvd + mdrd_175 + pth | 0.0069 | 0.0086 | 0.424 |
| calcium _\|\|_ phosphate \| + mdrd_175 + med_diur + pth | 0.0077 | 0.0086 | 0.374 |
| cvd _\|\|_ mg \| + age + mdrd_175 + med_diur | -0.0173 | 0.0291 | 0.553 |
| cvd _\|\|_ phosphate \| + diabetes + mdrd_175 | -0.0059 | 0.0274 | 0.829 |
| cvd _\|\|_ pth \| + mdrd_175 | 0.0005 | 0.0282 | 0.987 |
| diabetes _\|\|_ med_diur \| + cvd + mdrd_175 | 0.0437 | 0.0251 | 0.083 |
| diabetes _\|\|_ mg \| + age + mdrd_175 + med_diur | -0.0118 | 0.0275 | 0.668 |
| diabetes _\|\|_ mg \| + age + cvd + mdrd_175 | -0.0102 | 0.0276 | 0.713 |
| diabetes _\|\|_ pth \| + mdrd_175 | -0.0246 | 0.0265 | 0.355 |
| med_diur _\|\|_ phosphate \| + diabetes + mdrd_175 | 0.0519 | 0.0304 | 0.088 |
| med_diur _\|\|_ phosphate \| + cvd + mdrd_175 | 0.0563 | 0.0303 | 0.064 |
| med_diur _\|\|_ pth \| + mdrd_175 | 0.0220 | 0.0315 | 0.486 |
| mg _\|\|_ phosphate \| + diabetes + mdrd_175 | 0.0137 | 0.0070 | 0.052 |
| mg _\|\|_ phosphate \| + age + cvd + mdrd_175 | 0.0135 | 0.0070 | 0.055 |
| mg _\|\|_ phosphate \| + age + mdrd_175 + med_diur | 0.0135 | 0.0070 | 0.058 |
| mg _\|\|_ pth \| + mdrd_175 | -0.0022 | 0.0070 | 0.753 |

Implied conditional independencies stemming from the model were tested and refined the model until no gross violations were detected. We arrived at two possible adjustment sets. Both were used to obtain an adjusted estimate for the association between plasma Mg^2+^ and AAC. Creat, serum creatinine; cvd, cardiovascular disease; med_diur, diuretic medication use; Mg, magnesium; pth, parathyroid hormone.

**SUPPLEMENTARY FIGURES AND LEGENDS**

**
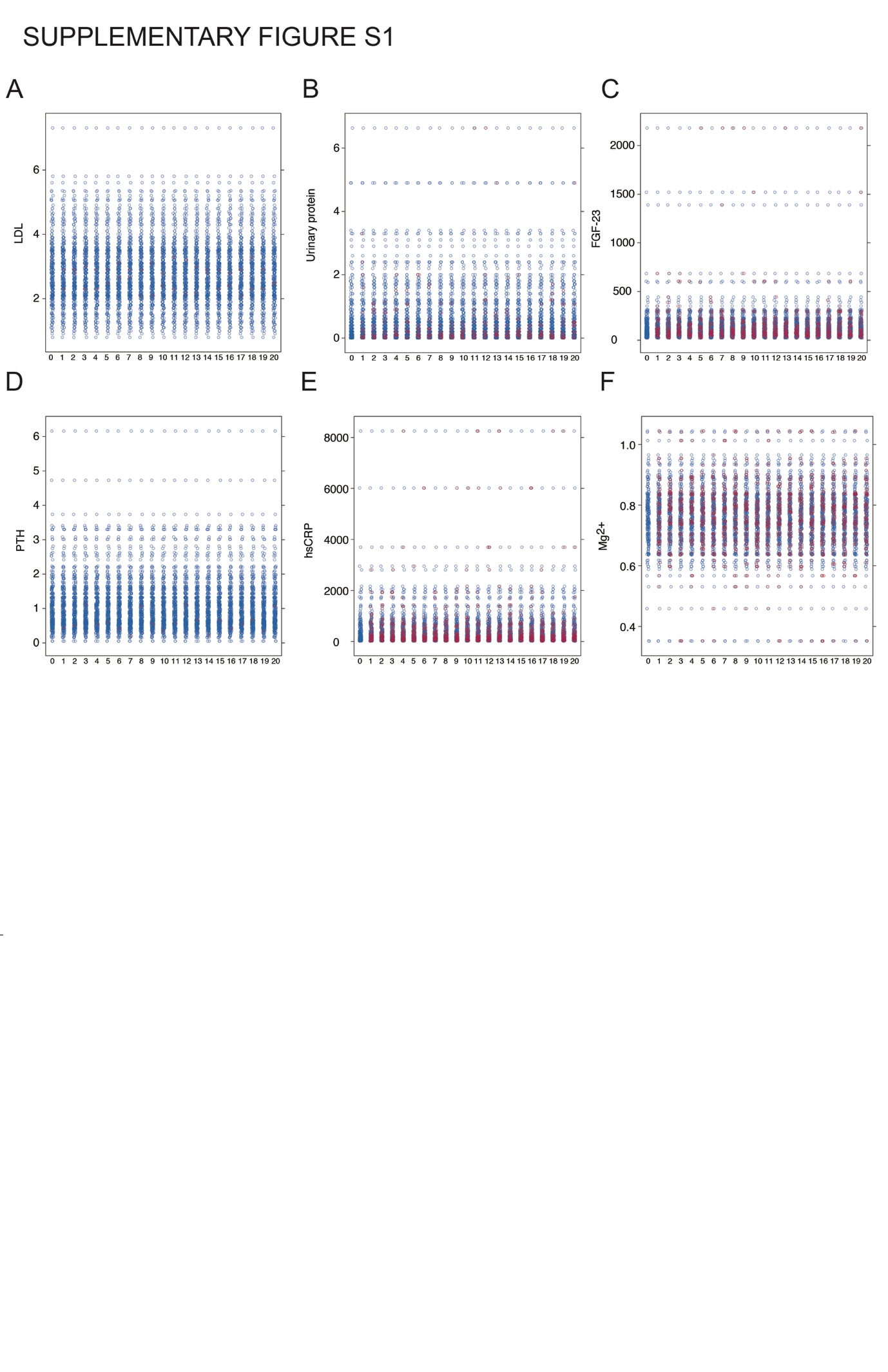
**

**Figure S1 Diagnostic strip plots.** Missing values were imputed using predictive mean matching. Predictor variables were selected based on bivariate correlation of at least 0.15. Measured and imputed values for baseline LDL cholesterol (A), proteinuria (B), FGF-23 (C), PTH (D), hsCRP (E) and Mg^2+^ (F), respectively. The x-axes show the imputation number with 0 being the unimputed, raw data. Known values are shown in blue, imputed values in purple. FGF-23, fibroblast growth factor-23; hsCRP, high sensitivity C-reactive protein; LDL, low-density lipoprotein; Mg^2+^, magnesium; PTH, parathyroid hormone.


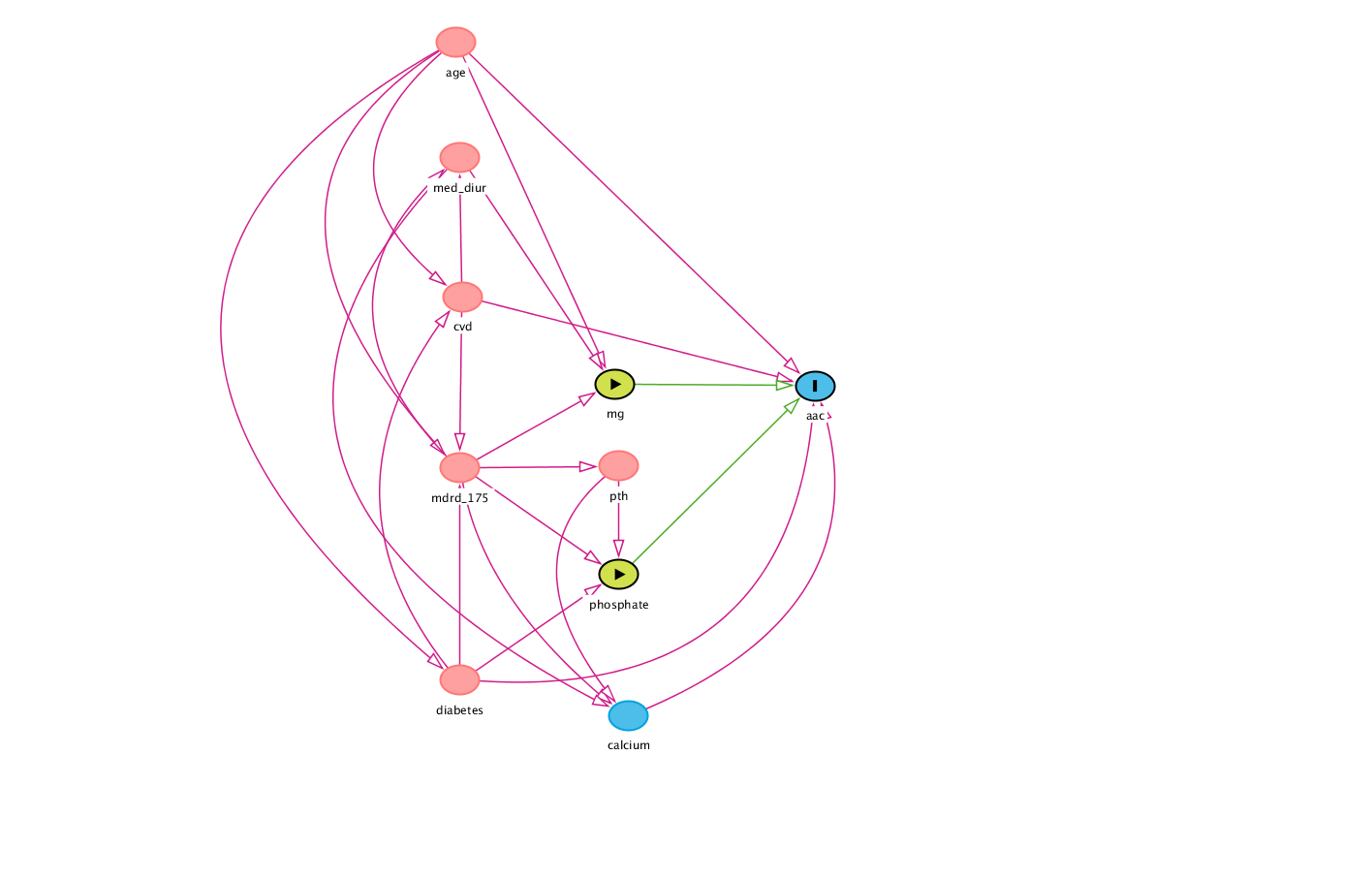


**Figure S2 Causal model for identification of adjustment sets.** Directed acyclic graph created with *dagitty.net* software and associated R-package to encode model assumptions. Variables depicted in the graph stem from literature research and are based on known relations with Mg^2+^, AAC or both. A final model was obtained by implying conditional independencies and pruning. Black symbols indicate ancestors of the outcome (blue symbol, AAC) and white symbols indicate ancestors of both the exposure (red symbol, Mg^2+^) and the outcome. Closed arrows indicate biasing pathways and open arrows indicate causal pathways. AAC, abdominal aortic calcification; Ca^2+^, calcium; CVD, cardiovascular disease; Mg^2+^, magnesium; Pi, phosphate; PTH, parathyroid hormone.

# **ITEM S1 SUPPLEMENTARY R-CODE**

## *Imputation of missing data*

# Set seed for replicability

set.seed(9820152)

# check missingness pattrns

md.pattern(dt.bas, plot = TRUE)

# Update prediction matrix

imp.bas <- mice(dt.bas, pred = quickpred(dt.bas, mincor = 0.15), m = 20, maxit = 0)

predmat.bas <- imp.bas$predictorMatrix

# Do not impute WHR and associated variables.

predmat.bas[c("waist", "hip", "whr"),] <- 0

predmat.bas[,c("waist", "hip", "whr")] <- 0

predmat.bas[, c("date_visit", "dob", "visit", "date_aac", "mdrd_175", "aac.bc")] <- 0

# Run the imputations (method = predictive mean matching)

imp.bas <- mice(dt.bas, pred = predmat.bas, m = 20, maxit = 5)

# Review the covergence

plot(imp.bas)

# Review the imputed values

stripplot(imp.bas, ldl)

stripplot(imp.bas, uprot)

stripplot(imp.bas, fgf23)

stripplot(imp.bas, pth)

stripplot(imp.bas, hscrp)

stripplot(imp.bas, cysc)

stripplot(imp.bas, mg)

Similar procedures were followed for the data at 1 year and at the time of X-ray.

## *Identification of the adjustment set*

dag.mg <- downloadGraph("dagitty.net/mQD1YaE")

plot(dag.mg)

condindep <- impliedConditionalIndependencies(dag.mg)

# create sest of lists to store results

result.localtest <- vector(mode = "list", length = length(condindep))

localtest <- vector(mode = "list", length = length(condindep))

# this loop obtains all the local tests (using linear regression) for all implied conditional independencies

for (j in seq_along(condindep)) {

# create a regression formula from the implied conditional independencies

x <- condindep[[j]]$X

y <- condindep[[j]]$Y

z <- condindep[[j]]$Z

eq <- paste0(x," ~ ", y)

for (i in seq_along(z)) {

eq <-paste0(eq, " + ", z[i])

}

# Perform the local test on the imputed data

localtest[[j]]<- with(imp.bas, lm(as.formula(eq)))

# description of the local test

lcltest.descr <- paste(x,"_||_",y,"|")

for (i in seq_along(z)) {

lcltest.descr <-paste0(lcltest.descr, " + ", z[i])

}

# Obtain the pooled result

result.localtest[[j]] <- cbind(lcltest.descr,summary(pool(localtest[[j]]))[2,])

}

d.localtest <- do.call(rbind, result.localtest)

rownames(d.localtest) <- NULL

# export the local test results

write.xlsx(d.localtest, file = "localtests_bas.xlsx", sheetName = "LocalTestsBas")

Similar procedures were followed for the data at 1 year and at the time of X-ray.

## *Zero-inflated Poisson model*

The mice packages had no automated procedures to implement the ZIP model. We performed the pooling as follows

# create a list with the data.frames at with baseline data, 1 year follow-up, and at time of X-ray.

dt.list.imp <- list(imp.bas, imp.1yr, imp.xray)

# Initialize a list to store results.

pooled <- vector(mode = "list", length = length(dt.list.imp))

# run the analysis for each of the data.frames.

for (i in seq_along(dt.list.imp)) {

# initialize the model

zip.ini <- with(complete(dt.list.imp[[i]], action = 1), zeroinfl(round(aac) ~ I(mg*10) + creat + calcium + cvd, , offset = I(time_aac/12))) # offset in years

# store parameter estimates for bootstrapping

ini.count <- dput(coef(zip.ini, "count"))

ini.zero <- dput(coef(zip.ini, "zero"))

run.zip <- function(x){

# Bootstrap SEs

zip.boot <- function(data, j) {

zip <- with(data[j,], zeroinfl(round(aac) ~ I(mg*10) + creat + calcium + cvd, offset = I(time_aac/12), start = list(count = ini.count, zero = ini.zero)))

as.vector(t(do.call(rbind, coef(summary(zip)))[,1:2]))

}

return(boot(complete(dt.list.imp[[i]], action = x), zip.boot, R = 100))

}

result.zip <- lapply(c(1:20), run.zip)

# Create a vector to store each of the parameters and the SEs

count.est <- vector(mode = "numeric", length = 20)

count.std.err <- vector(mode = "numeric", length = 20)

zero.est <- vector(mode = "numeric", length = 20)

zero.std.err <- vector(mode = "numeric", length = 20)

# Extract the parameters for each of the models created on imputed data

for (j in seq_along(result.zip)) {

count.est[j] <- summary(result.zip[[j]])$bootMed[3]

count.std.err[j] <- summary(result.zip[[j]])$bootSE[3]

zero.est[j] <- summary(result.zip[[j]])$bootMed[13]

zero.std.err[j] <- summary(result.zip[[j]])$bootSE[13]

}

# combine the estimates in a data.frame

zip.count <- as.data.frame(cbind(count.est, count.std.err))

names(zip.count) <- c("est", "std.err")

zip.zero <- as.data.frame(cbind(zero.est, zero.std.err))

names(zip.zero) <- c("est", "std.err")

# Apply Rubin's rules for pooled estimates

model <- list(zip.count, zip.zero)

zip.pooled <- function(x) {

# mean parameter estimate

q <- mean(x$est)
 # mean within imputation variance

u <- mean((x$std.err)^2)
 # between imputation variance

b <- sum((x$est-q)^2) / (nrow(x) - 1)

# total variance

t <- u + (1 + 1/nrow(x))*b

est <- q

se <- sqrt(t)
 # confidence limits

ll <- q - 1.96*sqrt(t)

ul <- q + 1.96*sqrt(t)

return(data.frame(est, se, ll, ul))

}

pooled[[i]] <- t(sapply(model, zip.pooled))

}

# merge the results for basline, 1 year, and at time X-ray into a single table

zip.adj <- do.call(rbind, pooled)

# transform log-odds to odds ratios

for (i in c(2,4,6)) {

zip.adj[i,]$est <- exp(zip.adj[i,]$est)

zip.adj[i,]$ll <- exp(zip.adj[i,]$ll)

zip.adj[i,]$ul <- exp(zip.adj[i,]$ul)

}

# export the result

write.xlsx(zip.adj, file = "zip.adjusted.xlsx")
